# Unrecognized and Undertreated Vertebral Fractures: What Else We Must Do

**DOI:** 10.1210/jendso/bvad139

**Published:** 2023-11-09

**Authors:** Sudhaker D Rao

**Affiliations:** Division of Endocrinology, Diabetes, and Metabolism, and Bone & Mineral Research Laboratory, Henry Ford Health, Detroit, MI 48202, USA

**Keywords:** vertebral compression fractures, fracture risks, osteoporosis, opportunistic diagnosis, medication trends, bisphosphonates, calcitonin

After more than a quarter century of progress in the diagnosis, detection, and management of osteoporosis, the initiation of therapy in real-world clinical practice remains quite low. Advances in basic and preclinical research led to well-designed and powered randomized clinical trials with clinically relevant endpoints and the approval of 10 effective drugs (antiresorptive and anabolic) to prevent and treat osteoporotic fractures. Several guidelines, position papers, and diagnostic algorithms have emphasized the need to curb this rising global health problem. Yet regrettably, the recognition of an obvious vertebral fracture (VF; by history or on imaging studies) and the initiation of antifracture therapy is dismal [1, 2]. As disappointing as it may seem, there are lessons to be learned and tasks to be implemented.

An osteoporotic fracture occurs every 30 seconds somewhere in the world and each fracture increases the risk of another fracture. Osteoporotic fractures are more common than strokes, heart attacks, and breast cancers combined [3]. Having a wrist fracture or VF doubles the risk of hip fracture, the most devastating of all osteoporotic fractures, with significant morbidity and mortality. Because of their anatomic location and immediate functional impairment, wrist and hip fractures are easily diagnosed and treated, but many VFs remain silent, with two-thirds detected incidentally, thus not recognized by the patient nor reported by the radiologists reading many clinical imaging studies [4, 5].

Here lies our challenge with VF detection. Unlike hypertension, diabetes, and hypercholesterolemia, which are detectable by physical or biological measures, detection of VFs requires a more focused approach, even though VFs are “plain in sight” of the reading radiologist. Retrospective studies have documented the low rate of reporting and suggested methods to improve recognition of VFs [4, 5]. A brief targeted training of primary care residents, for instance, on how to recognize VFs in imaging studies (eg, chest x-rays, computed tomography [CT] scans and magnetic resonance imaging [MRI] performed for unrelated reasons) improved the recognition of VFs [6].

In this context, the study by Malacon et al is informative, instructive, and illustrative. Using the “big data mining technique” the authors conclude that osteoporosis is underrecognized and undertreated following a low-energy “primary vertebral fracture” and thus the development of methods to decrease the risk of subsequent fractures is urgently needed [7]. The strengths of the study include a robust database and rigorous analysis, and a relevant target population that includes both commercial and governmental health care plans and covers all regions of the country. Most notably, while the VFs and anti-osteoporotic prescriptions increased from 2004 to 2019, the proportion of patients receiving treatment declined, most likely due to ill-perceived notions about the magnitude of rare adverse events of bisphosphonates. Only 11.7% of the patients >50 years started an anti-osteoporosis therapy [7]. Interestingly, calcitonin was the second-most common drug prescribed even though many already had existing VF who would benefit from more potent antiresorptive or even osteo-anabolic agents [7].

However, there are several limitations: First, the study was entirely based on ICD codes and claims data, which are subject to misclassification or missing data. Second, a paradoxical increase in VF rates in those receiving treatment even after accounting for potential confounders is surprising. This is probably a “noise” in the databases or that the patients who received treatment are inherently at a greater risk for subsequent fractures and needed more potent antifracture medications (bisphosphonates, denosumab, parathyroid hormone analogues, romosozumab), thus not necessarily “treatment failures.” I suspect that the third-party payers may have influenced the treatment modality decision than the clinicians, an area of great concern and in need of reform. Who should decide on the best therapeutic option for the patient with prevalent VF? Third, some concern about equity: younger patients, men, and blacks had lower treatment rates than other groups and the West region had the highest treatment rate. The authors attributed the latter to better socioeconomic, educational, and climatic conditions; not sure what this means for health care policy and reimbursement, but worth exploring. However, given the overall very low treatment rates in a presumably well-insured population, we can surmise that the treatment rate may be even lower for the underinsured and uninsured. Fourth, the delay in starting and the short time on treatment implies clinician inertia and poor adherence rate respectively. Finally, the database covers only a small fraction (19%) of the US patients covered by Medicare Advantage plans, thus representing a small subset of a large diverse population of the country. Nevertheless, the study calls for measures to improve recognition, detection, and management of osteoporosis. In a similar study of women with distal radial fracture, only 24% underwent either diagnostic evaluation or treatment of osteoporosis [8].

What else we must do? Just as cardiologists look at electrocardiograms, pulmonologists chest x-rays, and orthopedists skeletal x-rays, it is time to make sure all our colleagues start looking at x-rays (chest, CT, MRI, etc.) for an opportunistic detection of VFs before it is too late [4]. Radiologists, who are on the front line, make a concerted effort to routinely make a more direct note of the presence of VFs irrespective of the reason for the imaging study, at least in the at-risk population (>50 years of age for women and >65 years for men). Avoid ambiguous terms such as “wedging,” “endplate compression,” “endplate concavity,” and so forth, to indicate the presence of osteoporotic VFs [1]. This will not be an extra burden. The use of “smart phases” such as “incidental note is made of vertebral fracture, further evaluation suggested as appropriate” or some similar phrase would be a major first step. Reporting of incidental findings in radiology practice has been in existence for decades for a variety of findings (eg, lung nodules on chest x-rays, thyroid nodules on unrelated imaging studies, adrenal nodules on abdominal CT or MRI etc.), so why not same for VFs? Concern about possible malignancy should not be the only reason to report incidental findings. It is time we think of incidental VFs as “incidentalomas.”

## Disclosures

I have no conflicts of interest.

## Abbreviations

CT: computed tomography
MRI: magnetic resonance imaging
VF: vertebral fracture
